# A spatial analysis of dietary patterns in a large representative population in the north of The Netherlands – the Lifelines cohort study

**DOI:** 10.1186/s12966-017-0622-8

**Published:** 2017-12-07

**Authors:** Louise H. Dekker, Richard H. Rijnks, Dirk Strijker, Gerjan J. Navis

**Affiliations:** 1 Department of Nephrology, University Medical Center Groningen, University of Groningen, Groningen, The Netherlands; 2 Department of Economic Geography, Faculty of Spatial Sciences, University of Groningen, Groningen, The Netherlands; 3 Department of Cultural Geography, Faculty of Spatial Sciences, University of Groningen, Groningen, The Netherlands

**Keywords:** Dietary patterns, Spatial analysis, Environment, Food cultures

## Abstract

**Background:**

Diet is an important modifiable risk factor for chronic diseases. In the search for effective strategies to improve dietary patterns in order to promote healthy ageing, new approaches considering contextual factors in public health medicine are warranted. The aim of this study is to examine the spatial clustering of dietary patterns in a large representative sample of adults.

**Methods:**

Dietary patterns were defined on the basis of a 111 item Food Frequency Questionnaire among *n* = 117,570 adults using principal components analysis. We quantified the spatial clustering of dietary pattern scores at the neighborhood level using the Global Moran’s I spatial statistic, taking into consideration individual demographic and (neighborhood) socioeconomic indicators.

**Results:**

Four dietary patterns explaining 27% of the variance in dietary data were extracted in this population and named the “bread and cookies” pattern, the “snack” pattern, the “meat and alcohol” pattern and the “vegetable, fruit and fish” pattern. Significant spatial clustering of high (hot spot) and low (cold spot) dietary pattern scores was found for all four dietary patterns irrespective of age and gender differences. Educational attainment and neighborhood income explained the global clustering to some extent, although clustering at smaller regional scales persisted.

**Conclusion:**

The significant region-specific hot and cold spots of the four dietary patterns illustrate the existence of regional “food cultures” and underscore the need for interventions targeted at the sub-national level in order to tackle unhealthy dietary behavior and to stimulate people to make healthy dietary choices.

**Electronic supplementary material:**

The online version of this article (10.1186/s12966-017-0622-8) contains supplementary material, which is available to authorized users.

## Background

The burden of chronic diseases is rapidly increasing worldwide. Diet and nutrition are important factors in the promotion and maintenance of good health throughout the entire life course. Their role as determinants of chronic diseases is well established and they therefore occupy a prominent position in prevention and intervention activities [1]. In the search for effective strategies to improve diet in order to promote healthy ageing - and given the intuitively appealing notion that the food environment is an important determinant of diet - new approaches considering contextual factors, alongside the traditional focus such as individual determinants, in public health medicine are warranted [2, 3].

Traditionally, nutrition research has focused on single nutrients or specific foods, although individuals do not consume nutrients or foods in isolation. Thus, recent nutritional epidemiological studies have shifted to dietary pattern analysis, which describes the overall diet; the foods, food groups, and nutrients included; their combination and variety; and the frequency and quantity with which they are habitually consumed [4]. Focusing on dietary patterns, rather than individual nutrients or foods, can facilitate individual behavioral counseling and population dietary recommendations, because dietary patterns facilitate greater flexibility and personal preferences in dietary choices [5]. In addition, such patterns can lead to health benefits by means of smaller changes across several dietary factors, rather than major changes in a few factors, potentially increasing effectiveness and compliance to dietary recommendations. Insight in region specific dietary patterns may further aid the effectiveness and compliance as dietary change may be more readily achieved when recommended foods are compatible with existing patterns of food consumption.

Analysis of spatial clustering is an innovative way to examine the role of “place” as a contextual factor for dietary behavior, and may potentially inform (local) policy makers on targeting specific populations in need of dietary interventions. To our knowledge, there are no studies on the spatial clustering of dietary patterns. This is unfortunate as spatial analysis can offer exploratory and explanatory insights beyond traditional epidemiological approaches as they can be particularly sensitive to the selected scale of analysis, eliminating the limitation of predefined geographical units that may not coincide with empirically occurring regions. For example, analyses conducted using large geographical units such as provincial or state boundaries may not provide results at a resolution suitable for the development of local policies or decisions stimulating a healthy diet.

Spatial clustering techniques are commonly employed in conjunction with geographic information systems to explore whether the values of a variable follow a systematic spatial pattern. The search for positive—or negative—spatial clustering among variables is based on Tobler’s first law of geography which states that “everything is related to everything else, but near things are more related than distant things” [6]. Spatial analysis allows researchers to determine whether observations within a study area are random or exhibit a significant deviation from a pattern that would likely arise from random underlying factors. If a set of observations is not random, then it becomes important to measure the degree of the spatial distribution and to examine potential factors that may explain the clustering in order to make relevant hypotheses on its underlying cause.

Age, sex and socioeconomic status (SES) have been associated with measures of diet quality [7]. Geographically, these socio-demographic characteristics may differ according to the region of interest. For example in the Netherlands, there are more higher educated people living in cities as compared to the rural areas [8]. When dietary patterns are clustered geographically, it is important to examine whether SES – besides age and sex - may explain these spatial dependency.

We hypothesized that high or low adherence to empirically derived dietary patterns in a representative cohort of adults would cluster geographically. More specifically, neighborhoods with high (or low) adherence to a specific dietary pattern would be close to neighborhoods with a high (or low) adherence to that pattern, respectively. The present study examines the spatial clustering of dietary patterns derived by principal component analysis in the large population-based Lifelines Cohort Study taking into account geographical differences in SES.

## Methods

### Lifelines cohort study

Lifelines is a multi-disciplinary prospective population-based cohort study examining in a unique three-generation design to study the health and health-related behaviors of *n* = 167,729 persons living in the North of The Netherlands. It employs a broad range of investigative procedures in assessing the biomedical, socio-demographic, behavioral, physical and psychological factors which contribute to the health and disease of the general population, with a special focus on multi-morbidity and complex genetics. The Lifelines population is broadly representative for the people living in this region [9]. Detailed information on the cohort profile can be found elsewhere [10]. In brief, individuals living in the recruitment area aged between 25 and 50 years, were invited through their general practitioners. Individuals were not invited when the participating general practitioner considered the patient not eligible by reason of severe psychiatric of physical illness, limited life expectancy or insufficient knowledge of the Dutch language. In addition, inhabitants of the Northern provinces, who were not invited by their general practitioner and not meeting above-mentioned criteria, could register themselves via the Lifelines website. After signing to give informed consent, participants received a baseline questionnaire and an invitation to a health assessment at one of the Lifelines research sites. During these visits, participants were asked whether their family members would also be willing to participate. Overall, 49% of the participants (*n* = 81,652) were invited through their general practitioner, 38% (*n* = 64,489) via participating family members and 13% (*n* = 21.588) self-registered via the Lifelines website. Before study entry, all participants signed to give their informed consent. The Lifelines Cohort Study is conducted according to the principles of the Declaration of Helsinki and in accordance with the research code of the University Medical Center Groningen (UMCG).

### Food frequency questionnaire

We used a self-administered food frequency questionnaire (FFQ) to assess the habitual intake of 111 food items during the last month (4 weeks). An existing validated Dutch FFQ formed the basis for the FFQ used in the Lifelines study [11, 12]. The basic FFQ focused on estimates of energy intake and macronutrients, including alcohol intake. For 46 main food items, frequency of consumption was indicated as ‘not this month’ or in days per week or month, including the amount (in units or specified portion size) consumed each time. The FFQ also included 37 questions on consumption of sub-items (e.g. 20+/30+ cheese, 40+ cheese, 48+ cheese, or cream cheese) for which frequency was specified as never, sometimes, often and (almost) always. Values of nutrient contents of foods were obtained from the 2006 Dutch food composition Table [13].

For the present sample we included those Lifelines participants living in the three Northern provinces (Groningen, Drenthe and Fryslân) of the Netherlands who provided food frequency data (*n* = 128,147). To correct for potential under- or over-reporting on the dietary questionnaire, we excluded men and women who reported implausible energy intake (men <800 or >4200 daily calories; women <600 or >3500 daily calories). This resulted in *n* = 117,570 participants. The study has been approved by the medical ethics review committee of the UMCG.

### Dietary pattern analysis

Dietary patterns were derived on the basis of principal components analysis (PCA). PCA provides an informative picture of the linear correlation between types of foods consumed within a population. The coefficients defining these linear combinations are called factor loadings and represent the correlations of each food item/group with the dietary pattern. Since the proportion of explained variance per component (i.e. dietary pattern) decreases with the number of variables entered, individual food items with a similar nutrient profile and culinary use were combined into 33 food groups (See Additional file 1: Table S1).

Insight into these components (dietary patterns) may aid the development of nutritional intervention programs because it generates clear behavioral based dietary patterns commonly found within a (specific) population. In this exploration, the dietary patterns were derived on the basis of consumption (g/day) of each food group, unadjusted for energy intake. Within the PCA, orthogonal rotation (varimax option) was used to obtain uncorrelated patterns with greater interpretability. The decision to retain a component was based on the following grounds: component Eigenvalue >1.0 (indicating that the component explains more of the variance in the correlations than is explained by a single variable), identification of an inflection point in the Scree plot, and interpretability of the pattern.

Stability of the derived components was assessed by comparing the component solutions and factor loadings in two random halves of the data set and per sex group. A component was considered stable if the same major patterns were identified, meaning the food groups with significant contributions (factor loading >0.3 or <−0.3) were similar.

The factor score for each pattern was calculated by summing intakes of food groups weighted by their factor loadings. Factor scores were standardized to have a mean of 0 and standard deviation of 1. Scores reflect how closely a participant’s diet resembles each identified pattern, with higher scores representing closer resemblance.

### North of the Netherlands

Although according to some definitions the Netherlands contains no rural areas [14], the north of the Netherlands is generally considered a rural region [15]. The north of the Netherlands contains the three provinces Groningen, Fryslân, and Drenthe, with only three urban municipalities, based on the two highest categories of address density [8]: Groningen (provincial capital, in the maps: Gr), Leeuwarden (provincial capital Fryslân, in the maps Lw), and Assen (provincial capital of Drenthe, in the maps As). The north of the Netherlands is one of two main regions currently experiencing or anticipating population decline [16]. The main pockets of (anticipated) decline are along the mainland side of the Wadden Sea coast and the border with Germany.

Next to decline, the north of the Netherlands is also experiencing population ageing, with a combination of low birth-rates and out-migration of younger individuals contributing to an increase in numbers of retirees [17]. Finally, the three northern provinces lag behind economically, with only the province of Flevoland registering lower regional productivity and per capita income in the Netherlands [18].

### Neighborhood codes

Based on neighborhood codes [8], median factor scores were derived from the data based on *n* ≥ 5 participants per neighborhood, resulting in *n* = 1651 neighborhoods. From the total *n* = 116,447 participants from which data are mapped, the mean number of participants per neighborhood was *n* = 71 (median *n* = 29).

### Socio-demographic characteristics

Based on the participants’ responses to the self-administered questionnaires, data were assessed based on age, sex and educational -, and income level. Educational level was categorized in four categories (never been to school or elementary school only, lower vocational or secondary schooling, intermediate vocational schooling or intermediate/higher secondary schooling or higher vocational schooling or university). The level of income was defined by individual income categories and neighborhood income. Individual income categories were defined as a mean gross income of 1) < 1000 euro, 2) 1000–2000 euro, 3) 2000–3000 euro and 4) > 3000 euro. Neighborhood income (based on average individual income) was obtained from Central Bureau of Statistics [8].

### Statistical analysis

Univariate analysis was applied in order to assess the association between age, gender, educational level and dietary pattern scores. To produce dietary pattern score residuals, multivariate linear regression (ordinary least squares) was performed and dietary pattern scores were separately regressed on a set of hypothesized confounders of the relation between dietary patterns and neighborhood. Model 1 included age and sex and model 2 included model 1 + 1) educational level, 2) level of income or 3) mean neighborhood income. Dietary pattern residuals represent the portion of dietary pattern scores not explained by covariates in a model. Consequently, the spatial analysis of the dietary pattern residuals can be interpreted as clustering of low or high dietary pattern score variation not explained by the variables in the model.

### Spatial data analysis

The most commonly used global measure of spatial association is the Global Moran’s I (GMI) statistic [19]. The GMI statistic produces three different outcomes of spatial association. First, a significantly positive GMI means that areas with high values are found near other areas with high values, and low values near low values, more than would be expected given a random distribution. In this case, the conclusion is that spatial clustering takes place. Second, a significantly negative GMI means that areas with high values are more often found near areas with low values, and vice versa, than would be the case given a random distribution. This happens in the case of spatially competitive processes and is called dispersion. Third, a GMI that is not statistically significant means the null-hypothesis of a random distribution cannot be rejected, meaning that neither clustering nor dispersion takes place.

Global measures of spatial association identify whether clustering takes place at the level of the study area. There are two main limitations of global measures of spatial association. First, although global measures of spatial association can identify whether clustering takes place, they do not show where the high and low clusters occur. Second, using global measures of spatial association can fail to detect spatial patters if these only take place in part of the study area. The resultant global indicator of spatial clustering may come back as not significant because for the most part the data is randomly distributed (or even dispersed).

In order to overcome these limitations we use the Getis Gi* hot and cold spot analysis tool. The intuition behind the Gi* is that for each area i in the analysis, a weighted average is constructed for the variable under consideration, using the value for area i with a weight of 1, and the values of areas neighboring i, weighted by their distance to i. The resultant weighted average is normalized and can then be interpreted as a z-score of either a hot spot (positive z is indicative of higher values clustered nearer each other), or cold spot (negative *z* is indicative of lower values clustered nearer each other). These z-scores are then displayed in a map as either blue (cold spots) or red (hot spots) areas, and then shaded according to the *p*-values associated with this z-score (dark blue or red indicates a *p* < 0.01, medium blue or red indicates a *p* < 0.05, and light blue or red indicates a *p* < 0.10). The benefit of using the Gi* over other measures, such as the Local Moran’s I, is that the Gi*, being a weighted average, is less sensitive to local outliers which may result from spatial aggregation of the data.

For both the GMI and the Getis Gi* this study uses a bi-square nearest neighbor kernel, with the cut-off boundary set so that all regions have a minimum of one neighbor (6.7 km), with an inverse Euclidean distance weighting.

Age, sex and SES may be significant determinants of the dietary pattern scores and may potentially vary over the region. Therefore, the residuals from model 1 and model 2 were mapped and the Gi* was calculated.

## Results

The mean age of the participants in the Lifelines population is 45 years and 58% of the participants are women. Higher vocational schooling r university was attained by 31% of the participants, and the mean neighborhood income is 2124 euro. Most people in this cohort, 44%, life in an area with <500 addresses/km^2^ (Table 1).

**Table 1:** Population characteristics

Age (baseline) Mean (SD)		45 (13)
Sex, %	Female	58.4
Education, %	Never been to school or elementary school only	2.9
	Lower vocational or secondary schooling	27.9
	Intermediate vocational schooling or intermediate/higher secondary schooling	31.4
	Higher vocational schooling or university	37.7
Individual income (euro, %)	Unknown/no answer	14.7
	< 1000	6.8
	1000–2000	20.5
	2000–3000	28.5
	> 3000	29.5
Neighborhood income (mean, euro)		2124
Urbanization level by category, %^a^	1	6.7
	2	9.3
	3	15.6
	4	24.1
	5	44.3

^a^Level of urbanization: 1: very high > = 2500 addresses per km^2^; 2: high 1500–2500 addresses per km^2^; 3: moderate 1000–1500 addresses per km^2^; 4: low 500–1000 addresses per km^2^; 5: rural <500 addresses per km^2^

### Dietary patterns

By applying principal component analysis, we retained a four component solution based on an evaluation of the Scree plot of Eigenvalues and interpretation. These four components overall explained 26.6% (7.6%, 7.0%,6.4% and 5.6% respectively) of the variations in food intake and represent the four dietary patterns that commonly describe dietary behavior within this population. PCA conducted on the two random halves of the dataset yielded similar results (data not shown). The same four major patterns were identified for men and women, although the magnitude of the loadings differed more when compared to the derived outcomes in the random halves of the dataset (data not shown). The loadings of the food groups on the components (dietary patterns) are shown in Table 2. Positive loadings indicate that the subsequent food group is highly correlated with the corresponding dietary pattern, whereas negative loadings are inversely correlated. The first dietary pattern, which we labeled as the “bread and cookies” pattern, was characterized by high intakes of halvarine/margarine/butter, bread and bread products, sugar and confectionary, potatoes, cake and cookies, sauces/dressing/gravy, and high fat dairy products. The second pattern was labeled the “snack” pattern, and was characterized by high intakes of “other snacks”, pizza, ready to serve meals, French fries, sugar sweetened beverages, fruit/vegetable juices, sugar and confectionery, and low intakes of fruit. The third pattern was labeled as the “meat and alcohol” pattern, and was characterized by high intakes of fresh meat, processed meat, chicken, alcoholic drinks, coffee, sauces/dressing/gravy and low intakes of tea. The fourth pattern was labeled as the “vegetables, fish and fruit” pattern, and was characterized by high intakes of vegetables, fish and seafood, rice/pasta, legumes, fruit, nuts and seeds and eggs.

**Table 2:** Dietary patterns and factor loadings

	Bread and cookies pattern		Snack pattern		Meat and alcohol pattern		Vegetable, fruit and fish pattern	
	Food group and factor loadings^a^							
High intakes	Halvarine/margarine/butter	0.70	Other snacks	0.66	Fresh meat	0.60	Vegetables	0.57
	Bread and bread products	0.69	Pizza	0.55	Processed meat	0.59	Fish and seafood	0.50
	Sugar and confectionery	0.59	Ready to serve meals	0.60	Chicken	0.48	Rice/pasta	0.48
	Potatoes	0.53	French fries	0.48	Alcoholic drinks	0.45	Legumes	0.54
	Cake and cookies	0.44	Sugar sweetened beverages	0.46	Coffee	0.44	Fruit	0.41
	Sauces/dressing/gravy	0.43	Fruit/Vegetable juices	0.3	Sauces/dressing/gravy	0.38	Nuts and seeds	0.34
	High fat dairy products	0.36	Sugar and confectionery	0.39			Eggs	0.31
Low intakes			Fruit	−0.33	Tea	−0.45		

^a^Factor loadings | ≥ | 0.30

### Spatial autocorrelation and cluster identification

**Fig. 1:**
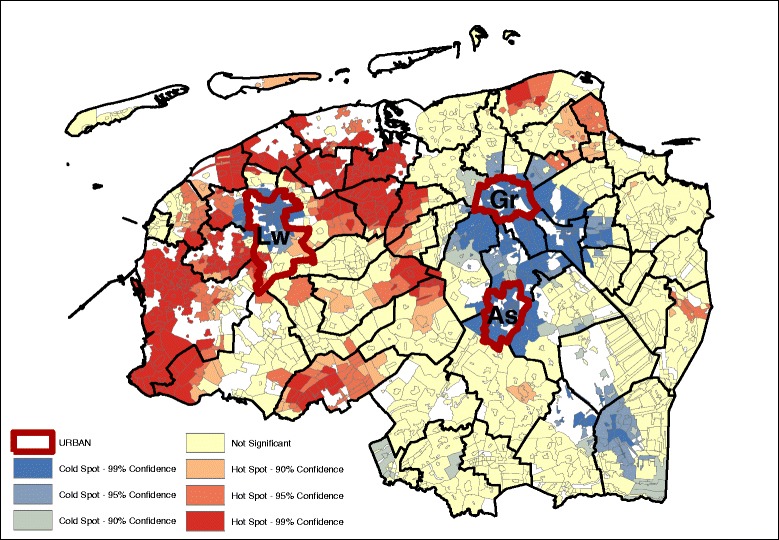
Bread and cookies pattern (GMI: 0.411, *p* ~ 0.00) – age, sex adjusted.

**Fig. 2:**
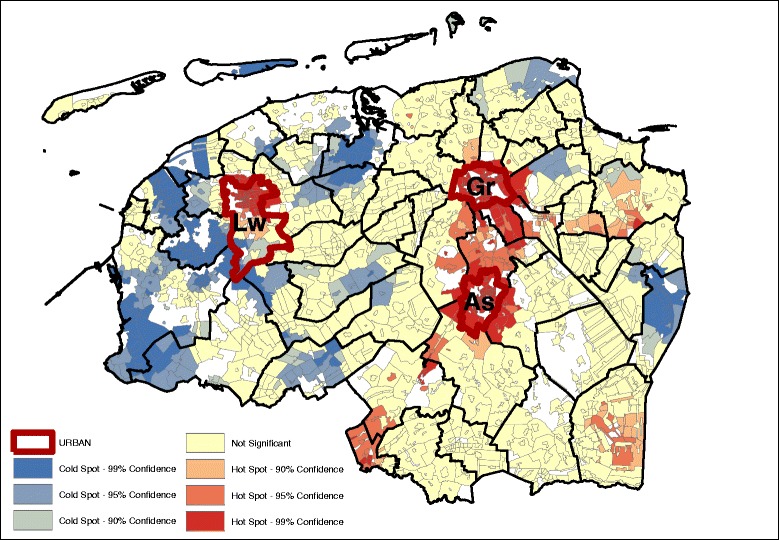
Snack pattern (GMI: 0.152, *p* ~ 0.00) – age, sex adjusted

**Fig. 3:**
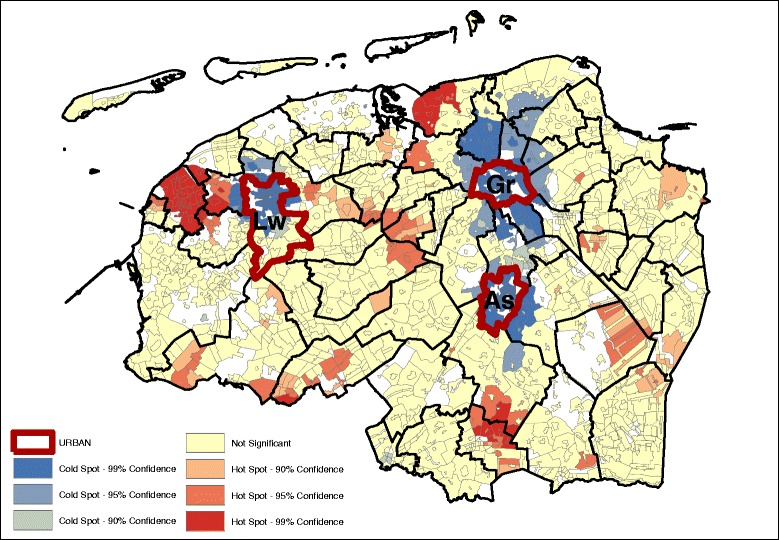
Meat and alcohol pattern (GMI: 0.235, *p* ~ 0.00) – age, sex adjusted

**Fig. 4:**
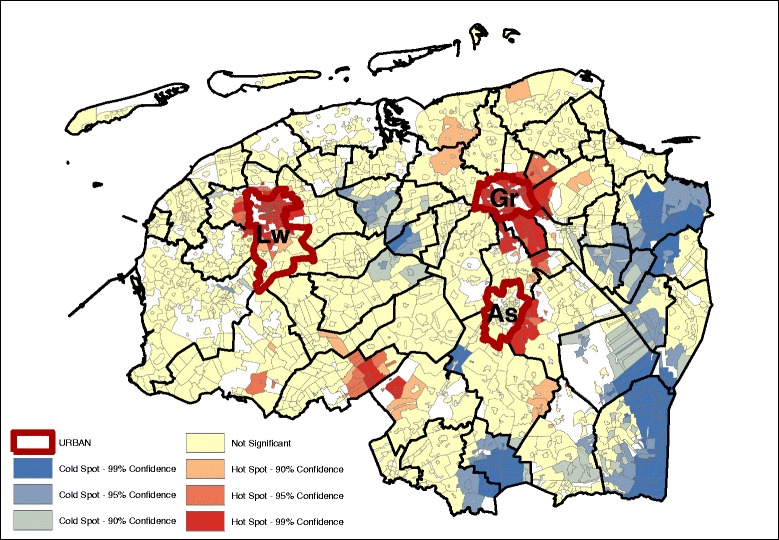
Vegetable, fish and fruit pattern (GMI: 0.126, *p* ~ 0.01) – age, sex adjusted

Significant age and gender adjusted spatial clustering was detected for the residuals at the level of the study area for the bread and cookies pattern (Fig. 1), snack pattern (Fig. 2), meat and alcohol pattern (Fig. 3) and the vegetable, fruit and fish pattern (Fig. 4). The values for the GMI’s are significant at the *P* ≤ 0.001 level (Additional file 2: Table S2), indicating that global clustering is observed with meaningful clusters of neighborhoods with relative high dietary pattern scores (hot spots), and on the other hand, clusters of neighborhoods with relative low dietary pattern scores (cold spots).

**Fig. 5:**
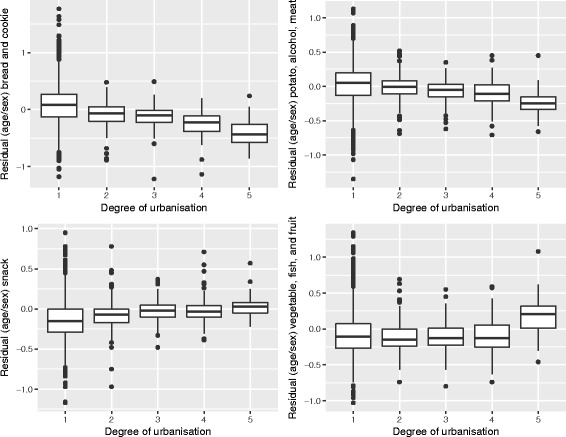
Age and gender adjusted dietary patterns by degree of urbanization

Geographically, we see that for the most part, the hot and cold spots correspond with the occurrence of urban centers. For legibility, the maps show the provincial capitals (municipalities), which are the only category 1 and 2 urban centers (at the municipality level) in the North of the Netherlands. The box and whiskers plot contain the category 1 and 2 urban centers at the neighborhood level (hence the higher number of regions). The box and whiskers plots in Fig. 5 show that the snack, and the vegetable, fruit, and fish dietary patterns are found mainly in the most urban areas (category 1), and less in the least urban areas (although not significantly less). Conversely, the bread and cookies pattern and the meat and alcohol pattern are consumed more in the rural areas, although the difference is again not significant.

Looking at the Gi* maps gives an indication of why these urban-rural differences are not significant. Neither areas classified as urban nor areas classified as rural are homogenous in their type of hot or cold spot. For example, the rural areas in the northern and western parts of the study area consume more bread and cookies, but this pattern is not observed in the south-east, while for the fruit, vegetable, and fish pattern the provincial capitals Groningen (Gr) and Leeuwarden (Lw) contain a hot spot but for Assen (As), the hot spot is just outside of the urban area. Second, in the case of the bread and cookies and the snack patterns, the spatial extent of the clusters surrounding the urban areas is much larger than just the urban areas alone. This means that, for instance, the lower intake of the bread and cookies pattern around the urban centers extends into the rural areas around the cities.

Table 3 gives an indication of the effect size difference between the dietary intake and their inclusion in hot or cold spots. The data displayed in the table are the mean dietary-adherence scores at the lowest level of spatial aggregation (median at the neighborhood level). Given that these are based on normalized dietary intakes, the size differences can be compared between the groups. The results indicate that the differences in adherence to the bread and cookies dietary pattern are most dispersed, with the mean adherence in hot spots (at the 95% confidence interval) with a range of 0.462. The meat and alcohol, and vegetables, fruit and fish dietary patterns show a much smaller difference between the hot and cold spots, with their ranges of 0.305 and 0.303 respectively.

**Table 3:** Median age and sex adjusted dietary pattern scores at hot and cold spots (*p* < 0.05)

	Hot spot	Neither	Cold spot
Dietary patterns	Dietary pattern scores		
Bread and cookies pattern	0.25	0.03	−0.22
Snack pattern	0.00	−0.12	−0.26
Meat and alcohol pattern	0.19	0.03	−0.14
Vegetables, fruit and fish pattern	0.10	−0.09	−0.23

### Socioeconomic status and spatial clustering of dietary patterns

**Fig. 6:**
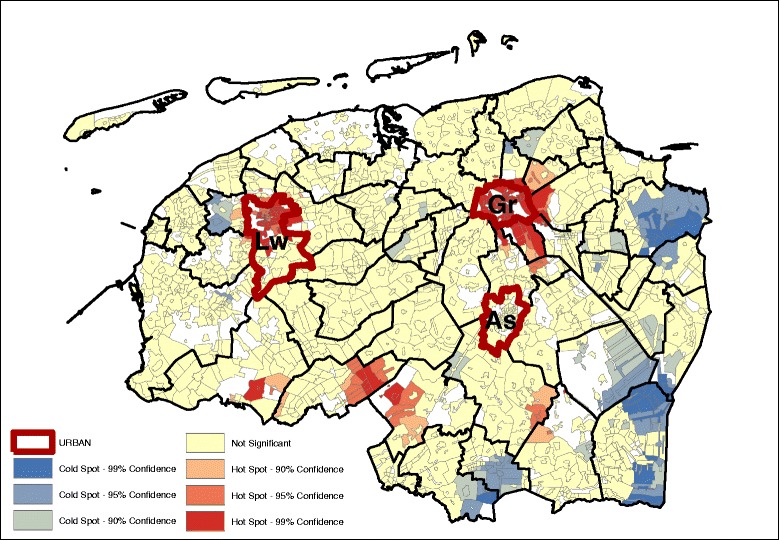
Vegetable, fish and fruit pattern (GMI: 0.060, *p* ~ 0.23) – age, sex, education adjusted

**Fig. 7:**
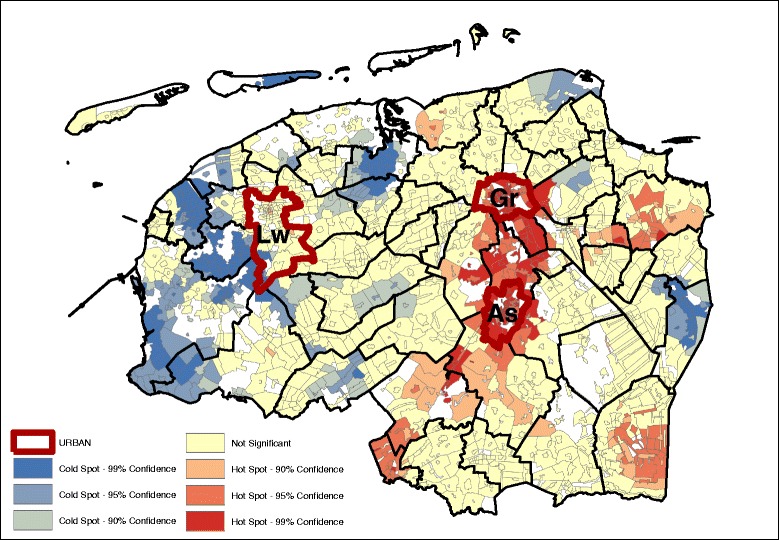
Snack pattern (GMI: 0.062, *p* ~ 0.22) – age, sex, neighborhood income adjusted

**Fig. 8:**
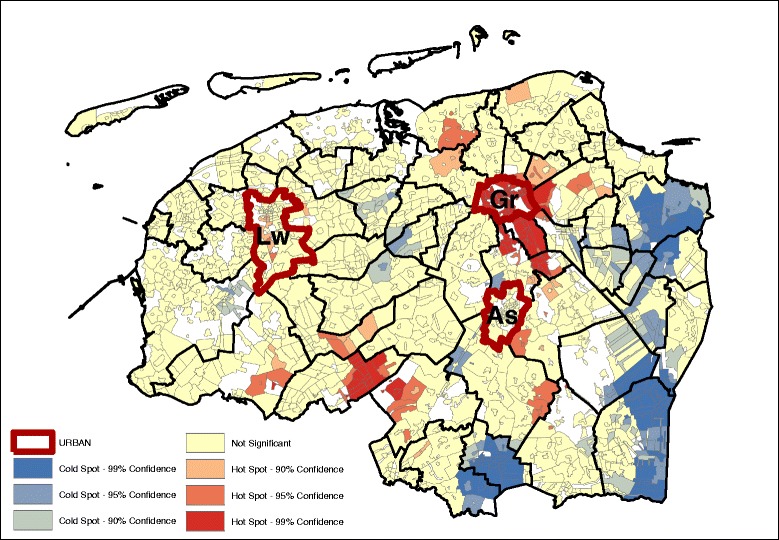
Vegetable, fish and fruit pattern (GMI: 0.017, *p* ~ 0.72) – age, sex, neighborhood income adjusted

Results from the OLS indicated that the level of educational attainment, individual income and neighborhood income were significantly associated with the dietary pattern scores (Table 4). Comparing the (degree of) spatial clustering based on the age and sex adjusted dietary pattern scores (Figs. 1, 2, 3, and 4) with the dietary pattern scores additionally adjusted for 1) education, 2) individual income or 3) neighborhood income, we observe some differences in spatial clustering based on the proxy of socioeconomic status (Additional file 3: Figure S1-S20). Educational attainment explains the spatial clustering of the vegetable, fish and fruit pattern (Fig. 6). For example, the hot spot near the city of Assen (As) has disappeared, and the cold spot along the eastern border has decreased in size. Although some hot and cold spot remain, the global measure of spatial clustering is not significant anymore (GMI = 0.06, *p* = 023). In contrast to individual income, which does not explain the spatial clustering for a four dietary patterns, adding neighborhood income to the model does influence to clustering. Taking into account differences in neighborhood income, the spatial clusters of the snack pattern (Fig. 7) and the vegetable, fish and fruit pattern (Fig. 8) disappears. The GMI that represent the spatial clustering on a global scale for all different models are presented in Additional file 2: Table S1. Despite the lack of clustering on the global scale for some patterns taking into account educational attainment of neighborhood income, the patterns of the Gi* still show the existence of local clusters of neighborhoods with relative high or low dietary patterns scores compared to the entire study area.

**Table 4:** Univariate associations between socio-demographic characteristics and dietary pattern scores

	Bread and cookies pattern	Snack pattern	Meat and alcohol pattern	Vegetable, fruit and fish pattern
	β (95% CI)	β (95% CI)	β (95% CI)	β (95% CI)
Age	−0.001* (−0.001;0.000)	−0.034** (−0.034;-0.033)	0.002** (0.001;0.002)	0.011** (0.011;0.012)
Gender	−0.56** (−0.57;-0.49)	−0.35** (−0.38;-0.35)	−0.71** (−0.72;-0.70)	−0.17** (−0.18;-0.16)
Educational level (4 levels)	−0.12** (−0.12;-0.11)	0.13** (0.12;0.14)	−0.10** (−0.11;-0.09)	0.15** (0.15;0.16)
Income (4 levels)	−0.21** (−0.02;-0.02)	0.02** (0.01;0.02)	0.04** (0.03;0.04)	0.055** (0.05;0.06)
Neighborhood income	−0.01** (−0.01;−0.01)	−0.01** (−0.01;-0.01)	-0.01** (−0.01;-0.01)	0.02** (0.02;0.02)

* *P* < 0.05, ** *P* ≤ 0.000

## Discussion

In the search for effective strategies to improve diet in order to promote healthy ageing at a resolution suitable for the development of local policies or decisions we explored the method of spatial clustering of empirically derived dietary patterns in a large representative sample in the Netherlands. A “bread and cookies” pattern, a “snack” pattern, a “meat and alcohol” pattern and a “vegetable, fruit and fish” pattern was derived from the Lifelines cohort. The adherence to these patterns was significantly spatially clustered although educational attainment and neighborhood income explained some of the spatial clustering.

While there is a growing body of literature exploring the influence of the built environment on both lifestyle and health outcomes [20–24], to our knowledge no analysis of spatial clustering of dietary patterns has yet been performed. The innovative finding that the adherence to dietary patterns is, to a large extend, spatially dependent provides an empirical basis for the development of (region specific) interventions. Based on the present results, interventions to promote a diet characterized by a high fruit, vegetable and fish intake may especially be warranted in the eastern part of Northern Netherlands, while in the larger cities interventions may be designed to stimulate a lower snack consumption. Instead of focusing on geographically defined boundaries, which may not necessarily correspond to the occurrence of health indicator under study, hot and cold spots that emerge from spatial analysis may provide insight into empirically occurring regions that may be in need of specific interventions.

The occurrence of cold spots with respect to the vegetable, fruit and fish pattern provides an empirical basis for in-depth research into potential barriers that may correspond with the occurrence of low intake of this presumably healthier dietary pattern. One such barrier is socioeconomic status.

In contrast to income, educational attainment explained some of the spatial clustering. This finding is in line with previous literature suggesting that education is a more important determinant of what could be described as a ‘healthy’ diet than income alone [25]. While individual income did not affect the spatial clustering, adjusted for neighborhood income on the other hand did change geographical distribution of some dietary patterns. For example based on the Global Moran’s I, the vegetable, fish and fruit pattern was not spatial dependent anymore. When we controlled for both individual income and neighborhood income, however, the spatial clustering remained significant for all dietary patterns. These results suggest that neighborhood socio-economic status can explain part of the clustering of food patterns, but that individual socioeconomic status should not be ignored. However, these results should be treated with caution, as combining individual data and regional data is associated with estimation problems, such as violating the independence of error terms and ecological fallacies [26].

Although previous spatial research has not focused on nutrition, studying spatial clustering of health related factors is not entirely new. Previous spatial analyses in the field of public health have mainly focused on clustering of BMI or overweight [27–31]. For example, in a study by Laraia et al. a significant level of clustering of extremely high and low BMI values among adults with diabetes was found. However, after adjusting for individual demographic and socioeconomic characteristics the spatial clustering disappeared, suggesting that individual factors accounted for most of the spatial autocorrelation at [27]. This is in line with a paper by Joost et al. in which the significant spatial clustering of BMI was explained by neighborhood income levels [29]. In a study by Huang et al., using individual-level data to detect obesity clusters, spatial concentration of obesity was wholly explained by neighborhood composition and socioeconomic characteristics [28]. Although closely linked, weight status and diet are two different health related concepts with different constructs. The finding that high or low adherence to dietary patterns significantly cluster at a regional level irrespective of differences in demographic and to some extend socioeconomic characteristics suggests the existence of regional dependent “food cultures”. In contrast to weight status, eating practices are related to deeply rooted cultural beliefs, perceptions and values [32], which intuitively are closely related to the region in which one is living. This may specially be true for regions with a low rate of “migration”, such as observed in this part of the Netherlands. The fact, however, that on a global scale no significant clustering appeared with respect to the vegetable, fruit and fish pattern, taking into account regional differences in age, gender and educational attainment, is in line with the grounded observation that healthier diets are, in general, consumed by better educated people [33].

This paper does not address the process by which these regional differences arise. However, there is an expanding body of literature dealing with external factors of dietary choice, through food availability research which investigates regional discrepancies in the availability, accessibility and affordability of e.g. fresh and healthy options [34–36]. In general, food environment is suggested to impact dietary behavior. However, at the neighborhood level, the evidence on the direction of effects is equivocal [37]. Some studies have shown that there are fewer healthy choices available in stores in deprived areas compared with less deprived areas [38] or that greater availability of neighborhood convenience stores was associated with lower diet quality [35], but others have shown few differences in healthy food availability between deprived and less-deprived neighborhoods [39, 40] or differences favoring more-deprived rather than less-deprived neighborhoods [41]. This possibility suggests that other factors may determine diet and questions whether it is really the region that is responsible for the “food culture”. More in depth studies on factors that are associated with dietary behavior is needed and the influence of the region alone are needed.

Besides external factors, a series of reviews point out that the evidence for important socio-cultural correlates of nutrition behaviors may be more convincing than that for physical environmental factors [37]. For example, results indicate that family socio-cultural factors such as parental encouragement and modeling are stronger correlates of intakes than availability of fruits and vegetables [42–44]. Looking at these internal factors of dietary choice, a recent comprehensive in-depth qualitative analysis on the role of socio-cultural traditions, beliefs and values with respect to food choices in Northern Netherlands suggests a differential role of specific family members in the food choices and different perceptions of food choices of different generations [45]. Investigating the processes underlying regional variations in consumption, both internal and external factors of choice should be synthesized. In-depth studies are warranted to examine the nature of associations of social roles and relationships, social institutions, social pressure and norms with respect to dietary behavior.

On a national scale, dietary recommendations such as the intake of sufficient fruits and vegetables, fish or abstinence in the consumption of sugar sweetened beverages, are poorly followed [46]. Although nationwide there would be a benefit from dietary interventions to enhance diet quality, spatial analysis of dietary patterns may provide an empirical basis on which to concentrate and target such interventions in those areas where the largest gains could be made. Our novel approach to assessing the adherence to dietary patterns at the neighborhood level may be beneficial in identifying areas of greatest intervention need. The significant region-specific hot and cold spots of dietary patterns underscore the need for interventions targeted at the sub-national level in order to tackle unhealthy dietary behavior and to stimulate people to make healthy dietary choices. This is in line with the current policy developments in several countries in Europe, such as the Netherlands and the UK in which the responsibility for public health is transferred to councils. This development has sparked a surge of innovative ways of looking at the role of “place” as an important determinant of health, such as diet. With regard to dietary surveillance the results from our exploratory analysis in combination with a hypothesis driven approach in which e.g. the compliance to dietary recommendations is examined may optimally encourage a step by step approach to stimulate healthy dietary behavior. Mapping lifestyle factors such as diet, in combination with other determinants of health such as wellbeing, health care utilization or socioeconomic status may further reveal potential “regions of opportunity” were efforts to increase public health as a whole may be most efficient. This may help local public health authorities to prioritize public health initiatives that may stimulate healthy ageing.

There are limitations related to the present study. First, dietary intake was based on self-reported data, and are subject to recall bias. However, we excluded participants with implausible energy intake and analyzed spatial clustering based on medians instead of means to eliminate the influence of outliers. Second, although PCA is extensively used in nutritional epidemiology and showed reasonable reproducibility and validity using FFQ data [47, 48], more validation studies are needed. PCA requires several arbitrary decisions, such as the pre-selection of food groups, the number of retained patterns, the method of rotation, and the cut-off value used to define a significant contribution of the factor loadings [49]. Yet, it was found that derived dietary patterns were robust for subjective factor analytical decisions. Third, dietary patterns derived by PCA are data driven and do not inform us on e.g. diet quality or compliance to dietary recommendations. An hypothesis driven approach combined with the methodology presented in this paper will optimally inform us on which populations in which regions are in need for a certain dietary intervention. Fourth, in order to safeguard the anonymity of the participants, the lowest resolution at which the location information was available was the neighborhood level, which limits the data used in this study to the median dietary adherence at the neighborhood level, with the minimum number of respondents per neighborhood of five respondents. The uses of the median diminishes the influence of outliers in potentially small subsamples per neighborhood. As the Gi* is a spatially weighted average, small-sample outliers could create spurious hot or cold spots. However, the use of aggregated data and measures of centrality means that some variance present in the raw data, and possibly spatial clustering, is lost in the process.

## Conclusions

In conclusion, the significant region-specific adherence to four dietary patterns in a large representative sample in the Netherlands illustrate the existence of regional “food cultures”. Although these food cultures can partially be explained by SES, the results still provide an empirical basis on the role of the “region” on dietary intake. A sub-national regional approach to increase healthy eating may potentially aid the effectiveness of and compliance to dietary recommendation as dietary change may be more readily achieved when recommended foods are compatible with existing patterns of food consumption.

## Additional files

Additional file 1: Table S1. Foods and food groups used in the dietary pattern analysis. (DOCX 30 kb)

Additional file 2: Table S2. Global Moran's I (GMI) and P value. (DOCX 27 kb)

Additional file 3: Figure S1-S20. Maps of all dietary patterns adjusted for age, sex, and neighborhood density, education, income or neighborhood income. (DOCX 2081 kb)

## Abbreviations

FFQ: Food frequency questionnaire
GMI: The Global Moran’s I
OLS: Multivariate linear regression
PCA: Principal components analysis
