# Supplementary material for: A spatial analysis of dietary patterns in a large representative population in the north of The Netherlands – the Lifelines cohort study
Source: Int J Behav Nutr Phys Act. 2017 Dec 7;14:166. doi: 10.1186/s12966-017-0622-8 (PMC5719934; doi:10.1186/s12966-017-0622-8)
Supplement: Supplementary file 1 — Foods and food groups used in the dietary pattern analysis. (DOCX 30 kb) [file 12966_2017_622_MOESM1_ESM.docx]

**Additional file 1 Table S1: Foods and food groups used in the dietary pattern analysis**

| **Food groups** | **Foods** |
| --- | --- |
| **Alcoholic drinks** | Beer, wine and fortified wine, white-, rosé and red wine, sherry, port wine or vermouth, spirits, distilled drinks (genever, whisky, rum, gin, cognac, vieux, liqueur) |
| **Bread and bread products** | Bread, crispbread, rusk, croissants and others |
| **Breakfast drink** | Breakfast drink |
| **Butter, margarine, low fat margarine** | Butter, margarine, low fat margarine |
| **Cakes and cookies** | Small cookies or (nutritional) biscuits, sponge cake, large cookies, cake, pie |
| **Cereals** | Muesli, granola or cereals for the preparation of porridges |
| **Chicken** | Chicken with and without skin |
| **Coffee** | Coffee |
| **Commercially prepared dishes** | Chinese/Indonesian dishes, meals from fast-food restaurants, other types of ready-to-eat meals |
| **Diet beverages** | Diet soft drinks or lemonade without sugar |
| **Eggs** | Boiled and fried eggs |
| **Fish and seafood** | Salted herring, fried herring, salmon, mackerel, eel, cod, plaice, haddock, pollack, sole, deep-fried whiting in dough etc. |
| **Fries** | Fried potatoes |
| **Fruit** | Fruit and apple sauce |
| **Fruit and fruit/vegetable juices** | Fruit and vegetable juices |
| **Savory bread toppings** | Savory bread toppings |
| **High fat milk products** | 40+ or 48+ cheese or spreadable cheese, cream cheese and/or foreign cheese, (full-fat) custard and other milk-based desserts, milk-based ice cream, Butter milk, full-fat plain yogurt, semi-skimmed plain yogurt, skimmed plain yogurt, chocolate milk, quark or fruit quark |
| **Legumes** | Brown beans, white beans, marrowfat peas, kidney beans etc. |
| **Low fat milk products** | 20+ or 30+ cheese or spreadable cheese, nonfermented medium/ low-fat milk or yoghurt |
| **Non-alcoholic drinks** | Alcohol-free beer |
| **Nuts and seeds** | Peanuts, nuts and seeds |
| **Other snacks** | Croquettes, minced meat hot dogs, sausage rolls, Savory snacks, potato chips or salty biscuits |
| **Pizza** | Pizza |
| **Potatoes** | Boiled or mashed potatoes |
| **Processed meat** | Luncheon meats, hamburger, minced meat (beef or mix of beef and pork), smoked sausages or frankfurters |
| **Red meat** | Sirloin steak, beef bratwurst, beef blade steak, beef rib steak or marbled beef, bacon, pork bratwurst, ‘slavink’ (ground meat wrapped in bacon), pork, beef steak, steak tartare, braising steak or roast beef |
| **Rice /pasta** | Pasta, rice |
| **Sauces** | Pasta sauce, mushroom sauce, sate sauce |
| **Sauces /dressing/gravy** | Regular mayonnaise, low fat mayonnaise, sauce for French fries and other non-red sauces, gravy, salad dressing with/without oil |
| **Soup** | Soups (with and without legumes) |
| **Sugar and confectionery** | Chocolate, candy with chocolate and chocolates, liquorice, acid drops etc., sweet sandwich toppings (chocolate- sprinkles, spread or flakes, honey, jam), sugar, honey or syrups, candy bars (Mars, Snickers, M&M's etc.) |
| **Sugar sweetened beverages** | Sugar sweetened beverages such as soft drinks (coke, orange flavored soft drinks, 7-up) or lemonade with sugar or sugar sweetened milk/yoghurt drinks |
| **Tea** | Tea |
| **Vegetables** | Vegetables |
