# Supplementary material for: A spatial analysis of dietary patterns in a large representative population in the north of The Netherlands – the Lifelines cohort study
Source: Int J Behav Nutr Phys Act. 2017 Dec 7;14:166. doi: 10.1186/s12966-017-0622-8 (PMC5719934; doi:10.1186/s12966-017-0622-8)
Supplement: Supplementary file 2 — Global Moran's I (GMI) and P value. (DOCX 27 kb) [file 12966_2017_622_MOESM2_ESM.docx]

| Dietary pattern | Adjustment | GMI score | P |
| --- | --- | --- | --- |
| Bread and cookie | AGE, SEX | 0.411 | 0.00 |
| Snack | AGE, SEX | 0.152 | 0.00 |
| Potato, alcohol, meat | AGE, SEX | 0.235 | 0.00 |
| Vegetable, fish and fruit | AGE, SEX | 0.126 | 0.01 |
| Bread and cookie | AGE, SEX, EDUCATION | 0.368 | 0.00 |
| Snack | AGE, SEX, EDUCATION | 0.136 | 0.01 |
| Potato, alcohol, meat | AGE, SEX, EDUCATION | 0.186 | 0.00 |
| Vegetable, fish and fruit | AGE, SEX, EDUCATION | 0.060 | 0.23 |
| Bread and cookie | AGE, SEX, INCOME | 0.400 | 0.00 |
| Snack | AGE, SEX, INCOME | 0.145 | 0.00 |
| Potato, alcohol, meat | AGE, SEX, INCOME | 0.237 | 0.00 |
| Vegetable, fish and fruit | AGE, SEX, INCOME | 0.121 | 0.02 |
| Bread and cookie | AGE, SEX, NEIGHBORHOOD INCOME | 0.129 | 0.01 |
| Snack | AGE, SEX, NEIGHBORHOOD INCOME | 0.062 | 0.22 |
| Potato, alcohol, meat | AGE, SEX, NEIGHBORHOOD INCOME | 0.098 | 0.05 |
| Vegetable, fish and fruit | AGE, SEX, NEIGHBORHOOD INCOME | 0.017 | 0.72 |

**Additional file 2: Tabel S2**
