# Supplementary material for: A spatial analysis of dietary patterns in a large representative population in the north of The Netherlands – the Lifelines cohort study
Source: Int J Behav Nutr Phys Act. 2017 Dec 7;14:166. doi: 10.1186/s12966-017-0622-8 (PMC5719934; doi:10.1186/s12966-017-0622-8)
Supplement: Supplementary file 3 — Maps of all dietary patterns adjusted for age, sex, and neighborhood density, education, income or neighborhood income. (DOCX 2081 kb) [file 12966_2017_622_MOESM3_ESM.docx]

**Additional file 3: Figures S1-S20**

Bread and cookie pattern

| Adjusted for: Adjusted for: Age, sex | Adjusted for: Age, sex, neighborhood density |
| --- | --- |
| 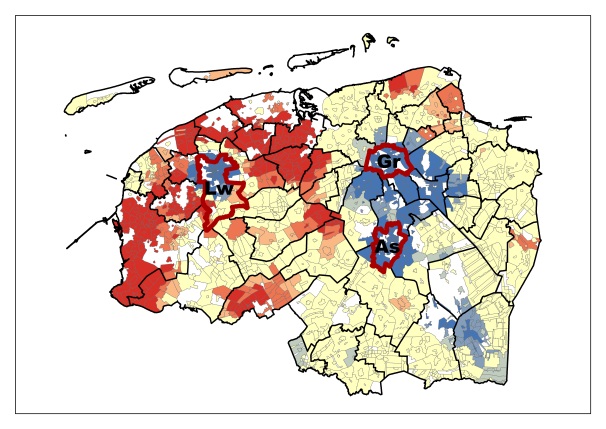 | 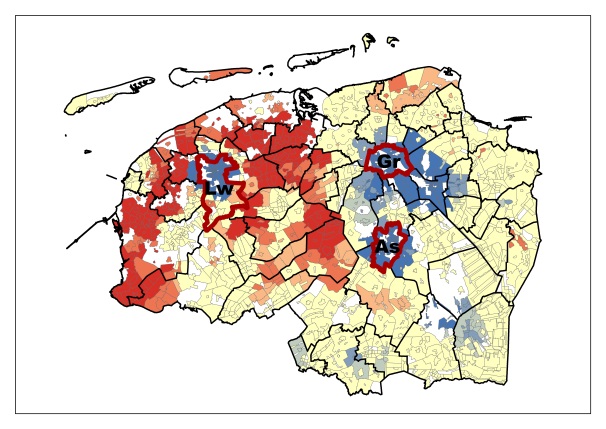 |
| Adjusted for: Age, sex, education | Adjusted for: Age, sex, income |
| 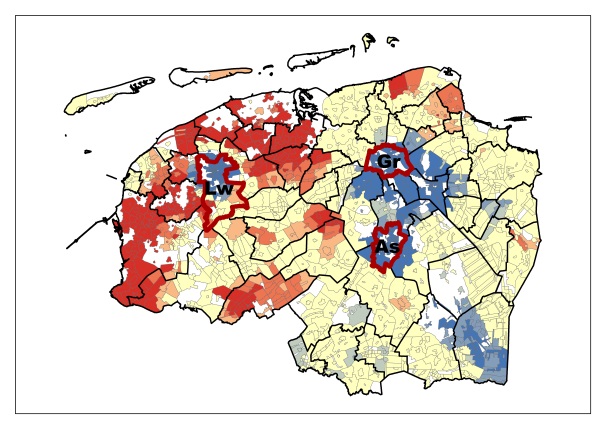 | 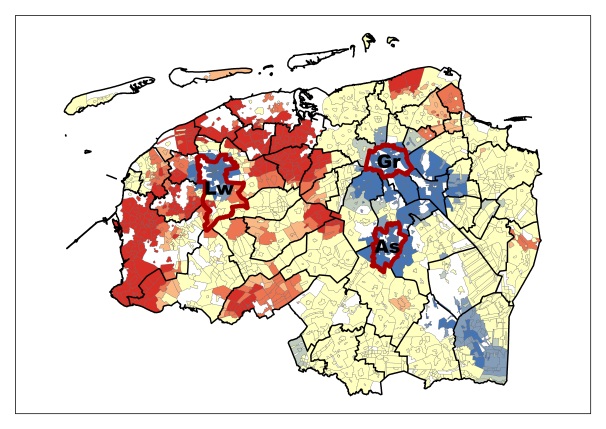 |
| Adjusted for: Age, sex, neighborhood income |  |
| 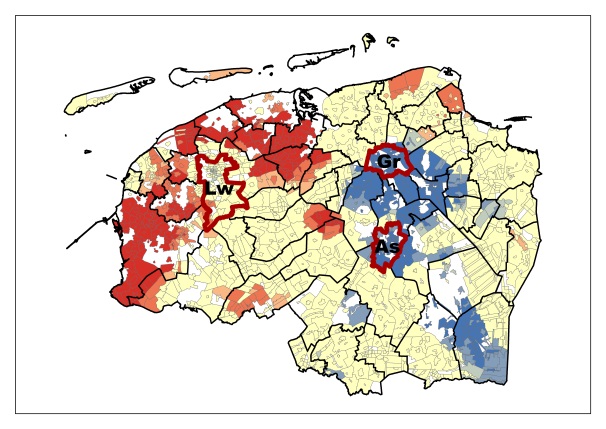 |  |

Snack pattern

| Adjusted for: Age, sex | Adjusted for: Age, sex, neighborhood density |
| --- | --- |
| 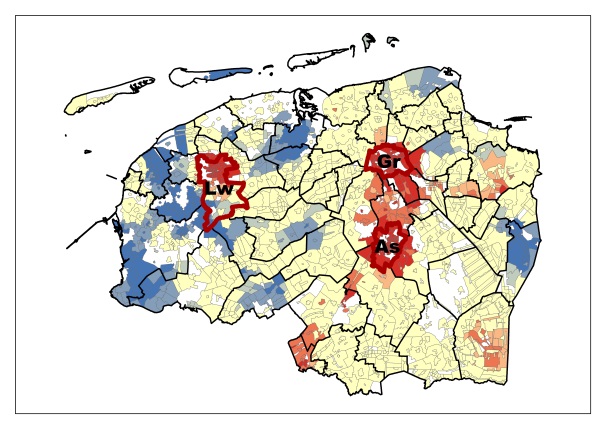 | 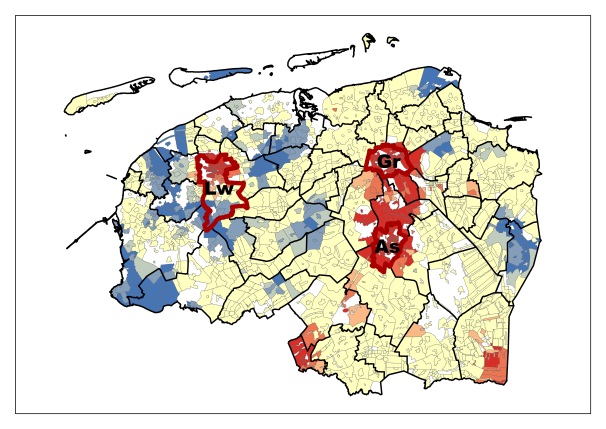 |
| Adjusted for: Age, sex, education | Adjusted for: Age, sex, income |
| 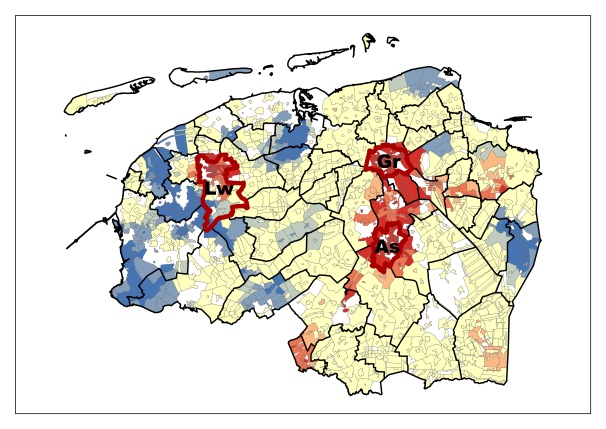 | 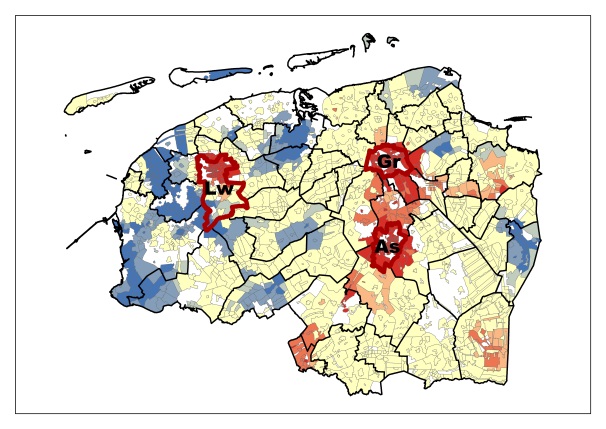 |
| Adjusted for: Age, sex, neighborhood income |  |
| 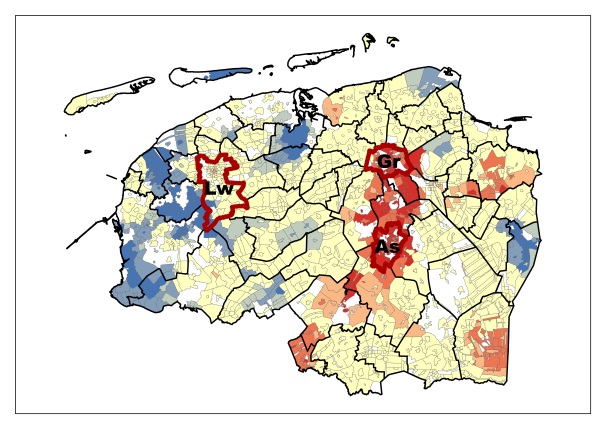 |  |

Potato, alcohol and meat pattern

| Adjusted for: Age, sex | Adjusted for: Age, sex, neighborhood density |
| --- | --- |
| 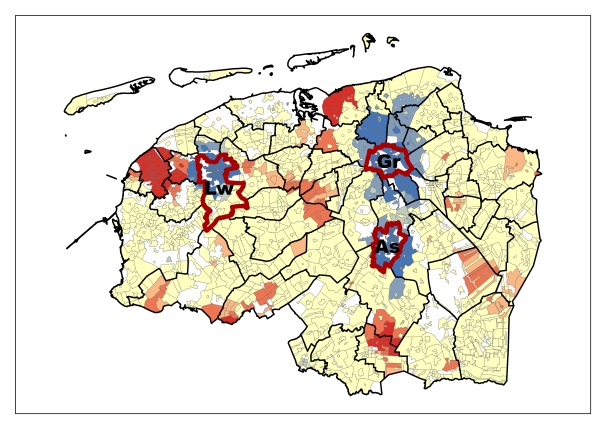 | 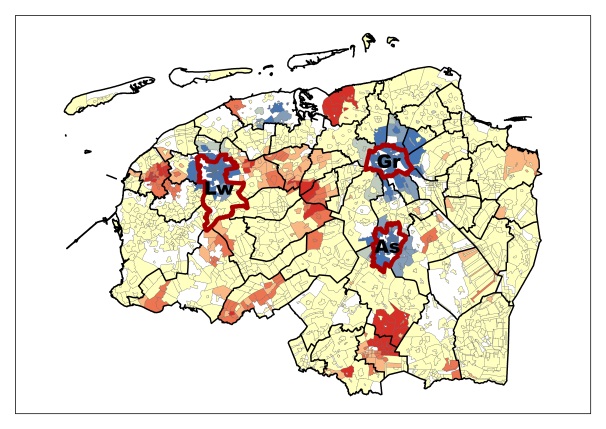 |
| Adjusted for: Age, sex, education | Adjusted for: Age, sex, income |
| 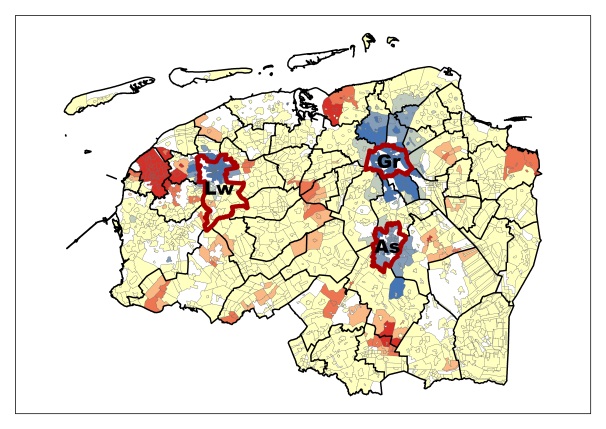 | 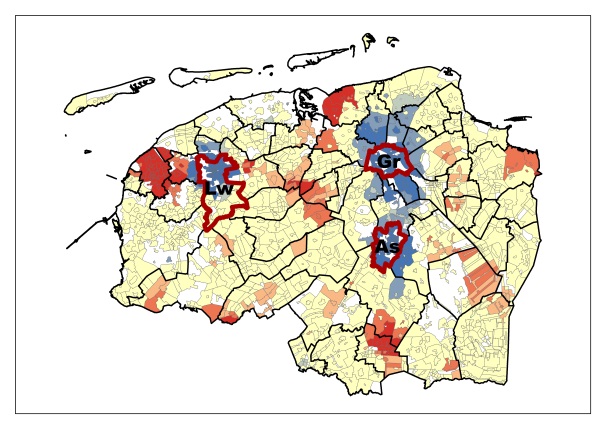 |
| Adjusted for: Age, sex, neighborhood income |  |
| 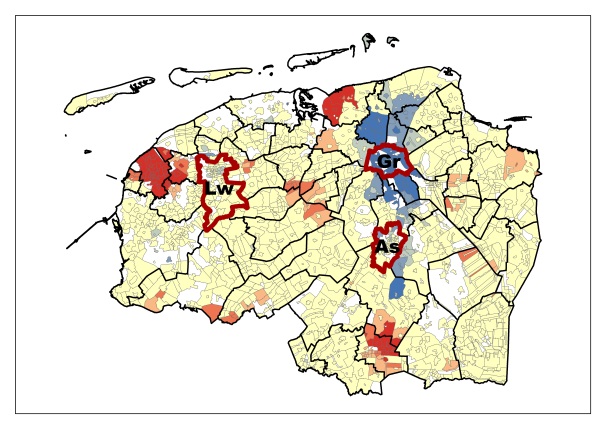 |  |

Vegetable, fis hand fruit pattern

| Adjusted for: Age, sex | Adjusted for: Age, sex, neighborhood density |
| --- | --- |
| 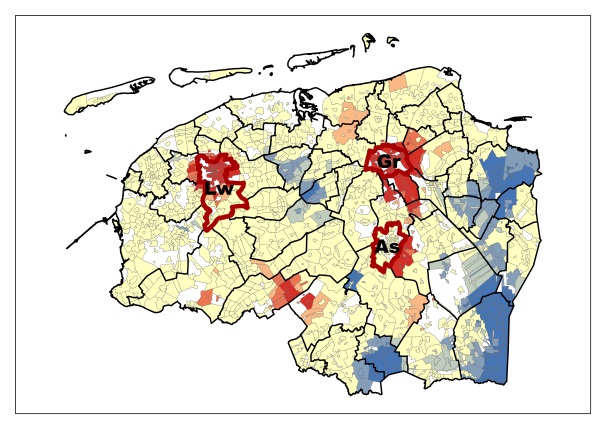 | 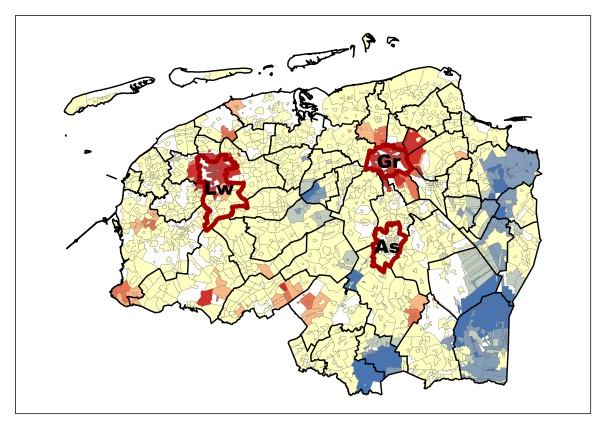 |
| Adjusted for: Age, sex, education | Adjusted for: Age, sex, income |
| 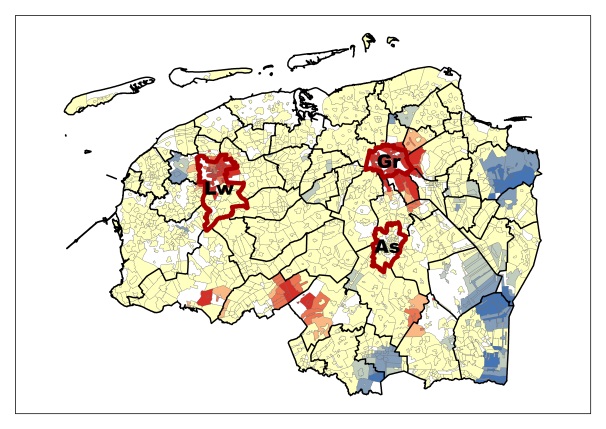 | 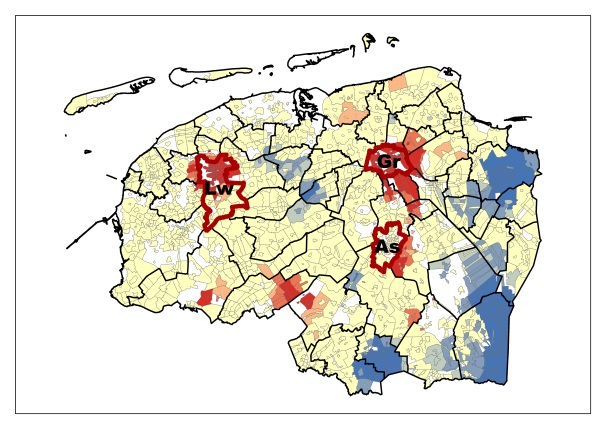 |
| Adjusted for: Age, sex, neighborhood income |  |
| 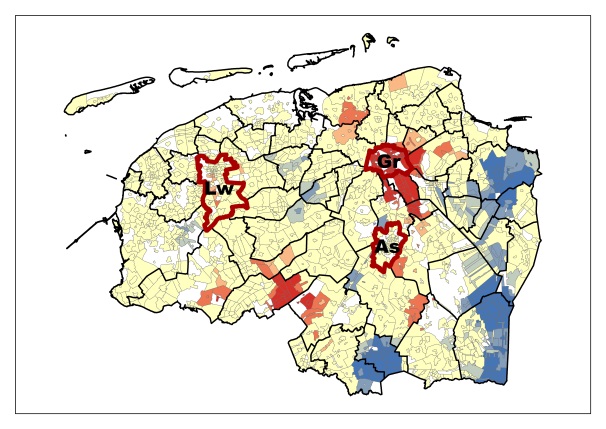 |  |
